# Maternal Allergy to Fluconazole—An Unusual Presentation: Case Report in a Breastfeeding Mother

**DOI:** 10.3390/children13030383

**Published:** 2026-03-09

**Authors:** Amani G. Ibrahim, Shajna Kinarullakandi, Badreldeen Ahmed, Justin C. Konje

**Affiliations:** 1 Feto Maternal Centre, Al Markhiya Street, Doha P.O. Box 2713, Qatar; 2 Obstetrics and Gynaecology, Qatar University, Doha P.O. Box 2713, Qatar; 3 Obstetrics and Gynaecology, Weill Cornell Medicine, Doha P.O. Box 2713, Qatar; 4 Obstetrics and Gynaecology, Department of Health Sciences, University of Leicester, Leicester LE1 7RH, UK

**Keywords:** neonatal thrush, antifungal, hypersensitivity reaction

## Abstract

**Highlights:**

**What are the main findings?**

- Hypersensitive reaction to a single dose of Diflucan administered as part of treatment of neonatal thrush.
- Angioedema, periorbital swelling, urticarial rash on face, neck, upper chest and lips.
- Rapid response to antihistamines.

**What are the implications of the main findings?**

- A thorough history should be taken before placing a mother on antifungal agents.
- Counselling should include possible allergic reactions.
- Treatment results in quick response and does not affect lactation.

**Abstract:**

**Background:** Allergy to fluconazole is uncommon and even more so in lactating women receiving treatment as part of treatment for neonatal oral thrush. **Methods:** We report a case of a rare and unexpected adverse reaction occurring after a single dose of fluconazole (Diflucan), administered to a mother of an exclusively breastfed neonate with oral thrush, as per guideline recommendations. **Results:** The woman developed multiple symptomatic manifestations highly suspicious of a hypersensitivity reaction after 8 h of taking a single 150 mg dose which were managed with antihistamines and cessation of medication. The baby’s symptoms abated with treatment and the mother whose hypersensitivity symptoms abated with treatment continued to breastfeed. **Conclusion:** We recommend that in prescribing antifungals to women whose babies have neonatal oral thrush, clinicians must always bear in mind that a history of previous allergic reaction(s) could signal the possibility of an allergic reaction to the antifungal agent. Counselling should therefore include the potential for reactions and steps to take if these develop. With supportive treatment, appropriate therapies and cessation of the medication, these invariably resolve. Where these persist, offering topical antifungal options may minimize the risk of recurrence of the hypersensitivity.

## 1. Introduction

Oral thrush, a fungal infection caused by the candida species, is very common in healthy full-term infants (4–15%) [1]. It is much more common in premature, immunocompromised, and antibiotic- or steroid-treated newborns, with rates reported to be as high as 37% [2]. Oral thrush typically presents as white, cottage-cheese-like plaques (patches) that cannot be easily removed on the gums, cheeks, or palate, and refusal of feeds. The affected babies usually cry inconsolably when attempting to feed from the breasts or the bottle and their nursing mothers may also have tender, inflamed and painful breasts and cracked or sore nipples [3]. Simultaneous treatment of both the mother and infant is essential to prevent recurrence. This is particularly important in exclusively breastfed babies as it ensures that feeding is not interrupted or stopped due to the risk of maternal mastitis or cracked nipples [4].

The first-line treatment of neonatal oral thrush is oral antifungal gels or creams for the baby and topical antifungal creams and oral fluconazole for the mother [5]. Fluconazole is an effective and potent azole-based antifungal agent which can have a range of reported adverse side effects (mild to severe) including gastric discomforts, headaches, rashes, Steven Johnson Syndrome, dizziness, ECG and liver function test abnormalities. Another of these adverse effects is allergy/hypersensitivity. While there have been reported cases in women on long-term therapy for vaginal candidiasis, we are not aware of a hypersensitivity reaction in a breastfeeding mother. Here, we report on a case of presumed hypersensitivity reaction to fluconazole in a breastfeeding mother and review the relevant literature. We then make recommendations on the management of such cases to ensure that breastfeeding is not disrupted.

## 2. Materials and Methods

### 2.1. Case Presentation

A healthy mother of a 6-day old girl (delivered by caesarean section and exclusively breastfed) presented to the Pediatric Clinic of the Feto-Maternal Centre, Doha Qatar with her baby who was refusing to breastfeed and had been crying inconsolably for five hours. The neonate who was clearly hungry was struggling to feed from the breast as well as with a nipple shield. She would release the breast after one gulp and start crying. The mother also complained of mild tenderness in one of her breasts. Efforts to supplement the newborn’s feeding with a milk formula or expressed milk from a bottle or a feeding syringe were unsuccessful.

Examination showed that the newborn had oral thrush (white sticky patches on the oral mucosa and under the tongue and whitish spots on the tongue), which was thought to account for her refusal of feeds and to breastfeed. The baby had no nappy rash and examination of the mother’s breast showed no signs of inflammation. Oral clotrimazole (Candizole) 3 times for 7 days was prescribed for the newborn and the mother given a single dose of fluconazole (Diflucan) 150 mg capsule (to be taken at night) and a topical antifungal cream (Ketoconazole cream) to apply on the breast 3 times a day for 10–14 days. The mother had never taken fluconazole before. She was instructed to wash off the cream before breastfeeding. The treatment of the baby and the mother followed standard NICE guidelines [5] for treatment of neonatal oral thrush.

Twenty-four hours after starting the baby on clotrimazole, her symptoms improved and the rash was less prominent, and she resumed breastfeeding satisfactorily. However, the mother, who had taken the oral fluconazole (Diflucan) just before going to bed, developed an urticarial rash on her face, neck and upper chest and lips, periorbital swelling, mild angioedema, palpitations and headaches—all starting approximately 8 h after taking the tablet. There were no associated systemic symptoms (e.g., cough, chest pain or breathing difficulties). By the time she was seen in the clinic, it was approximately 10 h from when she took the tablet. She had not taken any new food, medicine or drinks which could explain the hypersensitivity reaction. A presumed diagnosis of hypersensitivity reaction to fluconazole (Diflucan) was made based on the detailed timeline of the symptoms. She failed to attend the tertiary centre for an allergological evaluation. Previously, she had had a mild hypersensitivity reaction to an unspecified food item. She was placed on an oral antihistamine drug and advised not to apply the topical antifungal cream to her breast. The rash and itching improved within 24 h, and by 48 h, she had returned to normal.

### 2.2. Literature Review

A literature review was conducted using PubMed, CINAHL Embase, and the Cochrane Library. Searches covered from inception until September 2025 and used combinations of keywords and MeSH terms, including antifungal drugs, fluconazole, diflucan, breastfeeding, mother, hypersensitivity reaction, allergy and neonatal thrush/candidiasis.

## 3. Discussion

Fluconazole (Diflucan), used to treat a wide range of fungal infections, is generally well-tolerated. Allergic reactions are not uncommon, with a reported rate of 9.3 per 10,000 [6]—most occurring in patients who use it on a long-term basis. Most of these reactions are mild, although some can be severe enough to warrant hospital admission [6]. Kim et al., for example, reported on a 19-year-old woman who developed a generalized rash and eosinophilia after 30 days of fluconazole therapy for pulmonary coccidioidomycosis; despite these symptoms, she continued treatment and subsequently developed throat tightness and facial swelling, consistent with angioedema and a type I hypersensitivity reaction [7]. In another case report, a 25-year-old woman with a family history of atopic dermatitis who received monthly fluconazole for recurrent vaginal candidiasis developed erythematous macules after her second dose [8]. Demir et al. reported on a 45-year-old who developed multiple pruritic, erythematous, oval lesions on the trunk and extremities following her fourth dose of fluconazole [9]. Other studies have reported recurrent fixed drug eruptions [10] and extensive itchy maculopapular rashes which evolved into small vesicular lesions and crusting [11], appearing shortly after fluconazole administration for vaginal candidiasis. In one of these cases, there was a history of a previous drug allergy following recurrent administration of the drug (the rash developing after the third administration) [10]. In our case, the woman had also suffered from an allergy to an unknown substance (in her food), but this was mild.

While there are many documented allergic reactions to antifungal agents [6,12,13], these are more common in those receiving prolonged/repeated treatment for fungal infections. To the best of our knowledge, none of the previously reported cases were on a breastfeeding mother, and furthermore, on someone who had received a single 150 mg dose of fluconazole capsule as part of the treatment for oral thrush in her newborn. Our case is unique in that the patient developed a cutaneous hypersensitivity reaction after a single dose of fluconazole which was administered specifically for a breastfeeding-related indication. This highlights the need for clinicians and pediatricians to be aware of the fact that first-time exposure to fluconazole can cause hypersensitivity manifestations which could compromise breastfeeding in a mother–baby dyad where the baby is being treated for oral thrush. Fluconazole (Diflucan), a broad-spectrum triazole antifungal agent, generally considered a safe drug with few reported side effects contains multiple excipients—such as lactose, corn starch, colloidal silicon dioxide, magnesium stearate, and sodium lauryl sulfate (constituents that are found in some food items). Whether these constituents may contribute to the rare adverse reactions in susceptible individuals [11,14] is uncertain but we feel that some consideration should be given to them. In all the cases we reviewed, the reactions were consistent with a delayed Type 4 hypersensitivity reactions that occur hours after the use of the drug [12,15]. This was also likely the type of hypersensitivity in our patient. These reactions tend to be delayed, occurring after 48–72 h in most cases as they are mediated by T-cells and not antibodies, but in our case, the symptoms developed after 8 h, suggesting that previous exposure to the allergen might have taken place. Based on these observations, we would recommend that counselling about this possibility is considered, especially in those with a history of allergy, irrespective of how mild they are, as was the case in our patient. Furthermore, prompt intervention when this occurs should not have an impact on breastfeeding. In cases where the mother had previously been on long-term therapy for vaginal candidiasis and is then placed on oral antifungal agents because of an infected baby, we feel that this must also be discussed with advice to come for early treatment if symptoms develop.

In cases of hypersensitivity reaction to an antifungal drug, switching to an alternative is recommended, preferably after performing an allergological evaluation test. However, while such testing is ideal, it should not be the key determinant for switching as there is the recognition that allergic drug reactions can be diagnosed from a careful history, and where possible, incorporation of the Naranjo Adverse Drug Reaction Probability Scale to gauge the severity of the drug reaction [16]. The drug provocation test, which is the gold standard, is performed in hospitals, especially when there is need for the patient to take the drug frequently [15]. In one case, there was the need to continue Diflucan, and administering minute quantities led to desensitization, and to the patient finally receiving the full dose without experiencing the previous symptoms [17]. In cases such as ours, an alternative to Diflucan would be appropriate; however, caution must be exercised as there may be immediate or delayed side effects; hence, the switch should preferably be in hospital or under close monitoring. However, all the available antifungals (such as itraconazole, ketoconazole, miconazole) are azole-based and carry the potential for inducing allergic reactions [18,19]. Some studies suggest that not all cases of skin eruptions or rashes associated with fluconazole are allergic reactions to topical antifungal creams [20], but are rather induced by the systemically administered drug, which delivers higher and more prolonged drug exposure compared with topical application. Studies of single-dose 150 mg oral fluconazole for vaginitis/thrush have shown a higher incidence of side effects (26%) compared with intravaginal antifungal therapy (16%), highlighting that systemic administration carries greater risk of adverse outcomes [21].

## 4. Conclusions

Our case shows that even a single oral dose of fluconazole can provoke a hypersensitivity reaction in a previously healthy breastfeeding mother treated in the context of infant oral thrush. This highlights the need for clinicians to maintain vigilance when prescribing systemic antifungal therapy and to take a relevant drug history as well as ask about previous allergies and provide clear counselling regarding the possibility of allergic reactions. This may include advice to take the medication during the daytime and to seek immediate care at the emergency department if a hypersensitivity reaction occurs. Such a patient should be given appropriate therapies while being transferred to the emergency department. Diflucan alternatives might be considered in cases of resistance to topical antifungal treatments in nursing mothers with infants having oral thrush. An acknowledged limitation of our case was our inability to have an allergological evaluation to confirm the diagnosis. We, however, suggest that where this is available, it should be part of the long-term management.

## Data Availability

The original contributions presented in this study are included in the article. Further inquiries can be directed to the corresponding authors.

## References

[1] Stecksén-Blicks C., Granström E., Silfverdal S.A., West C.E. (2015). Prevalence of oral Candida in the first year of life. Mycoses.

[2] El-Dawy E.G.A.M., Baddar A., Gherbawy Y.A., Yassein A.S. (2025). Characterization and virulence of yeasts associated with neonatal thrush. BMC Microbiol..

[3] https://www.seslhd.health.nsw.gov.au/sites/default/files/groups/Royal_Hospital_for_Women/Mothersafe/documents/Factsheets/ThrushBfeedingFeb23.pdf.

[4] Johnstone H.A., Marcinak J.F. (1990). Candidiasis in the breastfeeding mother and infant. J. Obstet. Gynecol. Neonatal Nurs..

[5] National Institute for Health and Care Excellence (NICE) (2024). Clinical Knowledge Summaries: Candida—Oral.

[6] Shiferaw M.W., Donnelley M.A., Thompson G.R. (2025). Prevalence of antifungal allergies in the general population. J. Antimicrob. Chemother..

[7] Kim S., Chen K., Stull W. (2025). Fluconazole-induced drug rash with eosinophilia and systemic symptoms syndrome: A case report. J. Med. Case Rep..

[8] Tavallaee M., Rad M.M. (2009). Fixed drug eruption resulting from fluconazole use: A case report. J. Med. Case Rep..

[9] Demir S., Cetin E.A., Unal D., Coşkun R., Olgac M., Gelincik A., Colakoglu B., Buyukozturk S. (2018). Generalized Fixed Drug Eruption Induced by Fluconazole Without Cross-Reactivity to Itraconazole: Lymphocyte Transformation Test Confirms the Diagnosis. Drug Saf. Case Rep..

[10] Anooshiravani N., Nagarajan S., Vastardi M.A., Joks R. (2019). Inverse association of asthma and hay fever with cancer in the 2015 National Health Interview Survey database. Ann. Allergy Asthma Immunol..

[11] Di Leo E., Nettis E., Priore M.G., Ferrannini A., Vacca A. (2009). Maculopapular rash due to fluconazole. Clin. Exp. Dermatol..

[12] Ntoutoume Nzoghe P.C., Lakhmiri R., Coniquet S., Ntsame S., Hmamouchi I. (2026). Incidence of Adverse Drug Reactions at the University Hospital of Libreville, Gabon. Pharmacoepidemiology.

[13] Hernandez L.E., Jadoo A., Morrison B. (2023). Recurrent Fluconazole-Induced Fixed Drug Eruption of the Digit with Nail Matrix Involvement: A Case Report and Review of the Literature. Ski. Appendage Disord..

[14] Pfizer (2022). Diflucan: Package Information Leaflet; PIL/PK-02.

[15] Calogiuri G., Garvey L.H., Nettis E., Romita P., Di Leo E., Caruso R., Butani L., Foti C. (2019). Skin Allergy to Azole Antifungal Agents for Systemic Use: A Review of the Literature. Recent Pat. Inflamm. Allergy Drug Discov..

[16] Abrams E.M., Khan D.A. (2018). Diagnosing and managing drug allergy. CMAJ.

[17] Craig T.J., Peralta F., Boggavarapu J. (1996). Desensitization for fluconazole hypersensitivity. J. Allergy Clin. Immunol..

[18] Guliani A., Chauhan A. (2019). Fixed drug eruption due to itraconazole: A rare occurrence. Postgrad. Med. J..

[19] Bharija S.C., Belhaj M.S. (1988). Ketoconazole-induced fixed drug eruption. Int. J. Dermatol..

[20] Gomes T.F., Calado R., Matos A.L., Gonçalo M. (2022). Contact allergy to antifungals: Results of a 12-year retrospective study. Contact Dermat..

[21] Sobel J.D., Brooker D., Stein G.E., Thomason J.L., Wermeling D.P., Bradley B., Weinstein L., Fluconazole Vaginitis Study Group (1995). Single oral dose fluconazole compared with conventional clotrimazole topical therapy of *Candida vaginitis*. Am. J. Obstet. Gynecol..

